# Type I Diabetes Mellitus Increases the Cardiovascular Complications of Influenza Virus Infection

**DOI:** 10.3389/fcimb.2021.714440

**Published:** 2021-09-14

**Authors:** Jane E. Sinclair, Conor J. Bloxham, Han Chiu, Keng Yih Chew, Jake Russell, Yusuke Yoshikawa, Helle Bielefeldt-Ohmann, Lauren E. Steele, Katina D. Hulme, Nathalie AJ. Verzele, Ellesandra C. Noye, Melanie Wu, Melissa E. Reichelt, Walter G. Thomas, Linda A. Gallo, Meredith A. Redd, Kirsty R. Short

**Affiliations:** ^1^School of Chemistry and Molecular Biosciences, The University of Queensland, Brisbane, QLD, Australia; ^2^School of Biomedical Sciences, The University of Queensland, Brisbane, QLD, Australia; ^3^Institute for Molecular Bioscience, The University of Queensland, Brisbane, QLD, Australia; ^4^School of Veterinary Science, The University of Queensland, Brisbane, QLD, Australia

**Keywords:** cardiac, vascular, comorbid, type 1 diabetes mellitus, influenza A virus, *in vivo*, murine model

## Abstract

People with diabetes mellitus are susceptible to both cardiovascular disease and severe influenza A virus infection. We hypothesized that diabetes also increases risks of influenza-associated cardiac complications. A murine type 1 (streptozotocin-induced) diabetes model was employed to investigate influenza-induced cardiac distress. Lung histopathology and viral titres revealed no difference in respiratory severity between infected control and diabetic mice. However, compared with infected control mice, infected diabetic mice had increased serum cardiac troponin I and creatine-kinase MB, left ventricular structural changes and right ventricular functional alterations, providing the first experimental evidence of type I diabetes increasing risks of influenza-induced cardiovascular complications.

## Background

Influenza A virus (IAV) infection is associated with increased risk of cardiovascular disease (CVD) and heart attack, with acute myocardial infarction (MI) hospital admissions increasing six-fold in patients diagnosed with IAV in the preceding week (Kwong et al., 2018). The cause of IAV-associated CVD remains unclear. Direct IAV infection in the heart has been reported in both humans and mice (Davoudi et al., 2012). Alternatively, CVD and myocarditis may be an indirect outcome stimulated by circulating pro-inflammatory mediators, which also increase the risk of plaque rupture *via* local inflammation (Bazaz et al., 2013). Further contributing to intravascular complications, acute respiratory infections may trigger pro-coagulant and hemodynamic events and predispose the patient to secondary, perhaps intracoronary, thrombosis and ischemic cardiac disease, with enhanced leukocyte extravasation initiating atheroma formation (Bazaz et al., 2013).

Although the cardiac complications of IAV infection are now well established, the role of specific host co-morbidities in their development is unknown. Given the association of diabetes mellitus (DM) with both CVD (Giacco and Brownlee, 2010) and severe IAV (Reading et al., 1998), we hypothesised that DM is an important risk factor for cardiac complications from IAV infection. While clinical studies have recently been conducted on this topic, none differentiated between types 1 and 2 DM (T1- and T2DM), nor controlled for obesity (Samson et al., 2019; Chow et al., 2020; Modin et al., 2020), which we have shown to independently increase IAV-induced cardiac complications (Siegers et al., 2020). Here, we assessed whether DM contributes to increased IAV-associated cardiac impairment in T1DM IAV-infected mice.

## Methods

### Virus

A/Puerto Rico/8/1934(H1N1) stocks were prepared in embryonated chicken eggs and titers determined by Madin-Darby canine kidney (MDCK) cell plaque assay as previously described (Short et al., 2011; Brauer and Chen, 2015).

### Mice

All work was performed with ethical approval from The University of Queensland Office of Research Ethics (SBMS/071/17). Male C57BL/6 mice acquired from the Animal Resource Centre, Australia, were housed in individually ventilated cages under alternating 12-hour light/dark periods with food and water *ad libitum*. T1DM was induced using streptozotocin (STZ) (King, 2012); ten-week-old mice were fasted for four hours and intraperitoneally injected with 50mg/kg/day STZ (Sigma-Aldrich, Germany) or phosphate-buffered saline (PBS; control mice) daily for five days (King, 2012). STZ-treated mice were supplied with 10% sucrose water during, and until three days after, the injection period to prevent sudden hypoglycemia. DM was considered successfully established when non-fasting blood glucose concentrations (glucometer) were >16.7 mmol/L at one and two weeks post-injection (Zhu et al., 2005). All treated mice converted successfully. Both T1DM and control mice were randomized to intranasal inoculation with 1000 plaque-forming units (PFU) of A/Puerto Rico/8/1934(H1N1) IAV in 50µL, or PBS. Body weight was monitored daily and blood oxygen saturation was measured using a collar sensor and Mouseox Plus pulse oximeter (Starr, Oakmont, PA, USA).

### Echocardiography

Cardiac function was assessed at six days post-infection (d.p.i.) in all mice using the Vevo 3100 Imaging Platform (Fujifilm, Japan) with a 25-55 MHz transducer (MX550D). All measurements and analyses were conducted in a blinded manner. Mice were anesthetized with 2.5% isoflurane and general anesthesia maintained with 1% isoflurane during echocardiography. Mice in supine position were placed on a heating pad and heart rate and electrocardiography recorded. Body temperature was controlled and monitored for the duration of the echocardiography to maintain 37.0±0.5°C. Two-dimensional B-mode images were recorded in parasternal long axis view to determine ejection fraction (EF), stroke volume (SV), and cardiac output; and short-axis view to determine right ventricular (RV) fractional areas change (FAC). M-mode images were recorded in short-axis view to determine left ventricular (LV) end systolic and diastolic volumes, anterior wall thickness during systole and diastole (LVAW;s and LVAW;d), posterior wall thickness during systole and diastole (LVPW;s and LVPW;d), and mass, as well as RV free wall thickness (RVFWT); and in four-chamber view to determine tricuspid annular plane systolic excursion (TAPSE). Pulse-wave Doppler with color images were recorded in short-axis view to determine pulmonary acceleration time (PAT). Pulse-wave tissue Doppler images were recorded in four-chamber view to determine tricuspid and mitral early diastolic myocardial relaxation velocity (E’). CO, SV, and LV mass and volume during end systole and diastole were normalized to body weight [cardiac index, stroke volume index, LV mass index, LVESVI and LVEDVI, respectively]. While it was considered that normalizing to body weight when significant weight was lost over the course of infection might lead to overestimation of values indicating hypertrophy, as there was no significant difference between the final body weights of the infected diabetic and control group it was decided that this was acceptable. Recorded images were analyzed using Vevo LAB 3.1.1 software (VisualSonics, Toronto, Canada). Photographs exemplifying the analysis methods for different echocardiogram parameters are shown in **Supplementary Figure 1**. Parameters were measured three times per mouse in at least six mice and averages presented. Due to biosafety control measures, we were unable to measure and thus normalize to pre-infection baseline levels for each treatment group.

### Creatine-Kinase MB (CK-MB) and Cardiac Troponin I (CTNI) ELISAs

Serum levels of high sensitivity CTNI (Life Diagnostics, Inc., U.S.A.) and CK-MB isoenzyme (MyBioSource, U.S.A.) at six d.p.i. were determined according to the manufacturers’ instructions.

### Measuring Viral Titers *In Vivo*

Organs were harvested six d.p.i., homogenized *via* Qiagen Tissuelyser II (Qiagen, The Netherlands) and the supernatant isolated. RNA was extracted using NucleoZOL (BIOKÉ, The Netherlands) and viral cDNA synthesized using Oligo (dT)_18_ primers (Sigma-Aldrich, Germany). Viral copy number was determined by qPCR using IAV strain A/Puerto Rico/8/1934(H1N1) virus matrix (M) gene cloned into pHW2000 plasmid as described (Short et al., 2013). Viral titers were measured by MDCK cell plaque assay as described (Short et al., 2011).

### Histology

The left lung lobe was fixed in 10% neutral-buffered formalin, routine processed and embedded in paraffin, sectioned at 5μm and stained with hematoxylin and eosin. Sections were assessed for vascular changes, bronchitis, interstitial inflammation, alveolar inflammation, pneumocyte hypertrophy, and pleuritis by a veterinary pathologist blinded to the study design.

### Statistical Analysis

Data are pooled from two experiments, the initial containing six mice per group and the repeat containing 3 mice per group. Outliers were removed by ROUTS test (Q=1%). Normalcy was determined by D’Agostino-Pearson omnibus test or Shapiro-Wilk test. Body weight data were analysed using repeated measures three-way ANOVA, and other data were analysed using two-way ANOVA with *P*<0.05 indicating statistical significance. Where data was not normally distributed, statistics were performed on log-transformed data if this attained closer-to-normal distribution or untransformed data if transforming did not achieve closer-to-normal distribution, and presented as median ± IQR. Normally distributed data were presented as mean ± SEM.

## Results

### Pulmonary Severity of IAV Is Not Increased in Mice With T1DM

**Figure 1:**
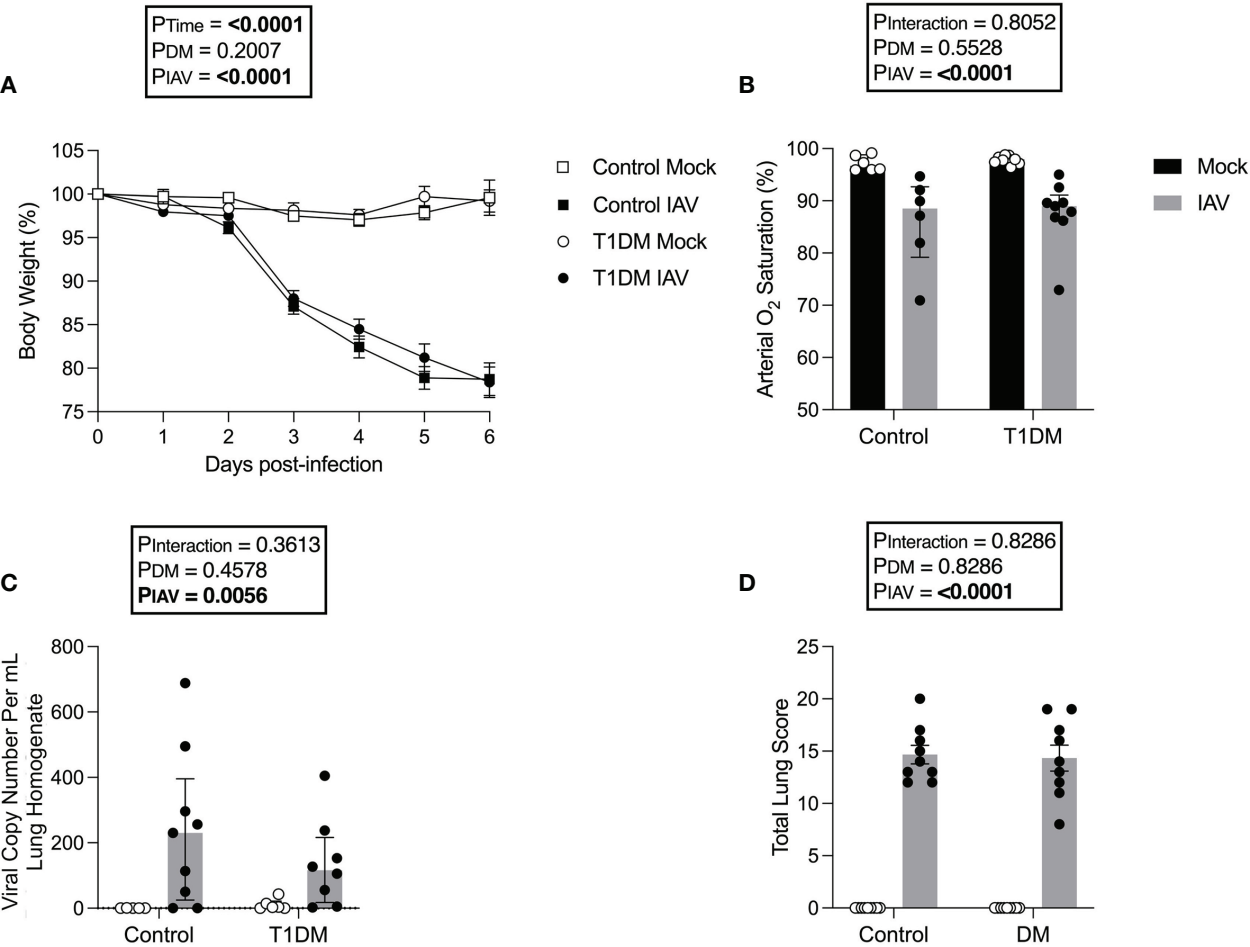
The pulmonary severity of IAV is not increased in T1DM versus control male C57BL/6J mice at six days post-infection. **(A)** Percentage of original body weight over infection course in IAV- or mock-infected mice with and without T1DM. **(B)** Arterial oxygen saturation percentage in IAV- or mock-infected mice with and without T1DM. **(C)** Lung IAV viral copy number in IAV- or mock-infected mice with and without T1DM. **(D)** Total lung histopathology scores in IAV- or mock-infected mice with and without T1DM. Data are pooled from two experiments, the initial containing six mice per group and the repeat containing 3 mice per group. Statistical outliers were removed by ROUTS test (Q=1%). For graph **(A)**, each data point represents the mean ± SEM of at least n=9 mice per group, for graphs **(B, C)**, each data point represents one mouse with median ± IQR of at least n=5 per group, and for graph **(D)**, each data point represents one mouse with mean ± SEM of at least n=9 mice per group. Statistical analysis was performed as described in “Methods”, being performed on untransformed data for graphs **(B, C)** as transforming did not achieve closer-to-normal distribution.

As obesity worsens the cardiac complications of IAV infection (Siegers et al., 2020), we used a well-established non-obese T1DM model to eliminate this confounding effect. Following IAV infection, there was significant weight loss in both control and diabetic mice but no significant difference was detected in weight loss between diabetic and control mice (**Figure 1A**). Similarly, IAV induced significant reductions in arterial oxygen saturation but there was no difference between diabetic and control infected groups (**Figure 1B**). At six d.p.i. no infectious virus was detected in the lung *via* plaque assay (data not shown due to lack of any plaques present) and the degree of pulmonary viral RNA was similar between diabetic and control groups (**Figure 1C**).

For all investigated lung histology parameters except pneumocyte hypertrophy/hyperplasia, IAV-infection increased pulmonary damage compared to mock-infection (**Supplementary Figure 2**). Diabetic mice had increased interstitial inflammation in comparison to control mice, with variance attributable to an interaction between IAV and DM (**Supplementary Figure 2**). However, total scoring found no significant difference in IAV-induced lung pathology between T1DM and control mice (**Figure 1D**).

### Markers of Cardiac Damage Are Elevated in IAV-Infected T1DM Mice

**Figure 2:**
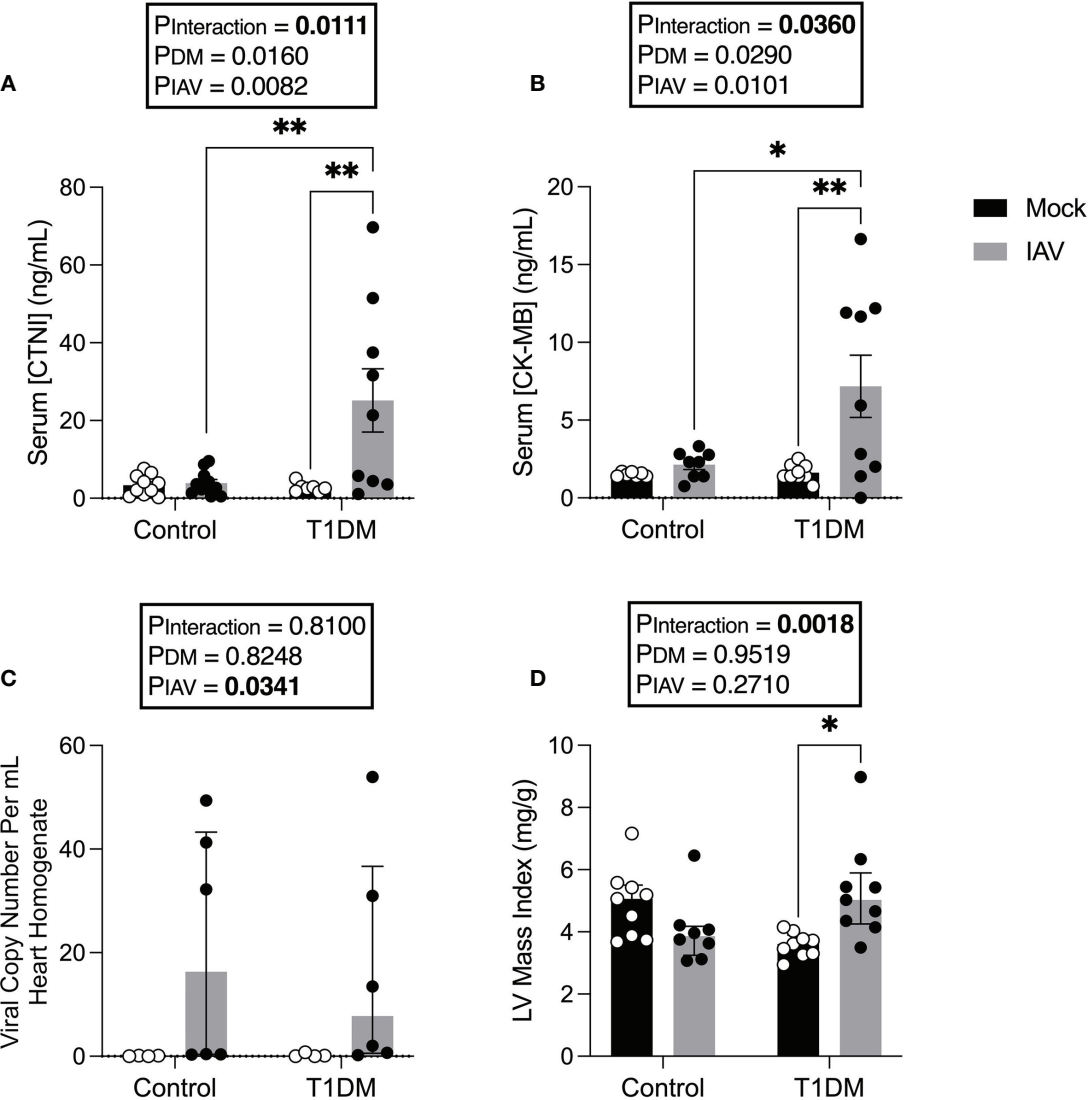
Increased markers of cardiac damage, and changes in left ventricular structure in IAV-infected T1DM versus control male C57BL/6J mice at six days post-infection. **(A)** Serum cardiac troponin I (CTNI) (ng/ml) in IAV- or mock-infected mice with and without T1DM. **(B)** Serum creatine kinase MB (CK-MB) (ng/ml) in IAV- or mock-infected mice with and without T1DM. **(C)** IAV viral copy number present in hearts of IAV- or mock-infected mice with and without T1DM. **(D)** Echocardiogram-derived left ventricular mass index in IAV-infected mice with and without T1DM. Data are pooled from two experiments, the initial containing six mice per group and the repeat containing 3 mice per group. Statistical outliers were removed by ROUTS test (Q=1%). Each data point represents one mouse with at least n=6 per group and mean ± SEM for graphs **(A, B)**, and median ± IQR for graphs **(C, D)**. Statistics were performed on log-transformed data for LV mass index as these showed the closest to normal distribution, but on untransformed data for graph **(C)** as transformation did not achieve closer-to-normal distribution. Statistical analysis was performed as described in “Methods” with *P<0.05; **P<0.01.

We next sought to assess whether there was evidence of increased CVD in T1DM mice with IAV, despite the absence of increased respiratory disease. IAV infection significantly increased serum CTNI and CK-MB in T1DM, but not in control, mice (**Figures 2A, B**).

To assess whether these data reflected virus replication in the heart of T1DM mice, viral titres in the heart were assessed. No infectious virus was detected in the hearts of any of the treatment groups (data not shown). Whilst low levels of viral RNA were detected in the hearts of infected mice, this was not significantly different between IAV-infected T1DM and control mice (**Figure 2C**).

### IAV Alters Cardiac Structure and Function in T1DM Mice

To investigate whether IAV infection and DM had detrimental effects on cardiac function, echocardiograms were performed. At six d.p.i. we observed changes in cardiac structure and function (**Table 1**). Functionally, IAV decreased cardiac index within the LV. In the RV, IAV decreased PAT, the magnitude of tricuspid E’ and TAPSE. Structurally, IAV increased LVPW;s. DM increased LV stroke volume index and cardiac index. IAV and DM interactions were attributable for a significant increase in LV mass index in T1DM mice only upon IAV infection (**Figure 2D**).

**Table 1:** Echocardiogram parameters assessing cardiac function in mice.

	Control		Type 1 DM		Findings		
	Mock (n=9)	IAV (n=9)	Mock (n=9)	IAV (n=9)	DM	IAV	Interaction
**Global Parameters**							
Heart rate (bpm)	478.5 ± 18.74	384.9 ± 19.04	460.9 ± 20.71	351.6 ± 12.82	*p=*0.1684	***p*<0.0001**	*p=*0.6643
**Functional status**							
**Left ventricle**							
Cardiac index (mL/min/g)	0.455 ± 0.057	0.285 ± 0.040	0.536 ± 0.056	0.464 ± 0.058	*p*=**0.0198**	*p*=**0.0290**	*p*=0.3643
EF (%)	61.562 ± 4.120	61.324 ± 3.472	67.027 ± 3.958	67.656 ± 3.472	*p*=0.1272	*p*=0.9590	*p*=0.9092
Stroke volume index (uL/g)	0.941 ± 0.094	0.800 ± 0.123	1.145 ± 0.085	1.352 ± 0.165	*p*=**0.0038**	*p*=0.7898	*p*=0.1597
Mitral E’ (mm/s)	-14.896 ± 2.460	-16.687 ± 1.490	-16.759 ± 2.186	-15.710 ± 0.709	*p*=0.8165	*p*=0.8457	*p*=0.4586
**Right ventricle**							
FAC (%)^a^	47.94 ± 13.11	63.15 ± 14.66	65.96 ± 11.3	59.08 ± 30.65	*p*=0.1721	*p*=0.3672	*p*=0.1465
PAT (ms)	15.555 ± 1.021	10.092 ± 0.728	14.443 ± 0.972	12.686 ± 1.018	*p*=0.4375	*p*=**0.0006**	*p*=0.0580
TAPSE (mm)	0.482 ± 0.056	0.357 ± 0.033	0.522 ± 0.078	0.365 ± 0.036	*p*=0.6621	*p*=**0.0135**	*p*=0.7607
Tricuspid E’ (mm/s)	-14.395 ± 1.823	-6.687 ± 0.843	-14.180 ± 2.202	-8.737 ± 1.263	*p*=0.5837	*p*=**0.0004**	*p*=0.4994
**Structural status**							
**Left ventricle**							
LVAW;s (mm)^b^	1.542 ± 0.274	1.461 ± 0.260	1.376 ± 0.433	1.573 ± 0.448	*p*=0.8284	*p*=0.8260	*p*=0.6660
LVAW;d (mm)	1.00 ± 0.065	0.836 ± 0.062	0.793 ± 0.054	0.862 ± 0.071	*p*=0.1413	*p*=0.4188	*p*=0.0651
LVPW;s (mm)	1.07 ± 0.061	1.267 ± 0.092	1.178 ± 0.041	1.492 ± 0.136	*p*=0.0787	*p*=**0.0090**	*p*=0.5241
LVPW;d (mm)	0.766 ± 0.055	0.820 ± 0.029	0.801 ± 0.035	1.096 ± 0.177	*p*=0.1331	*p*=0.0935	*p*=0.2387
LV mass index (mg/g)^b^	5.066 ± 1.697	3.859 ± 0.928	3.611 ± 0.623	5.027 ± 1.643*	*p*=0.9519	*p*=0.2710	*p*=**0.0018**
LVESVI (uL/g)	0.743 ± 0.105	0.476 ± 0.065	0.576 ± 0.066	0.595 ± 0.076	*p*=0.7605	*p*=0.1276	*p*=0.0811
LVEDVI (uL/g)^b^	1.959 ± 1.006	1.481 ± 0.318	1.786 ± 0.608	2.061 ± 1.005	*p*=0.4593	*p*=0.3953	*p*=0.1978
**Right ventricle**							
RVFWT (mm)	0.402 ± 0.040	0.463 ± 0.038	0.532 ± 0.062	0.515 ± 0.039	*p*=0.0613	*p*=0.6439	*p*=0.4112

EF, ejection fraction; E’, early diastolic myocardial relaxation velocity; FAC, fractional area change; PAT, pulmonary acceleration time; TAPSE, tricuspid annular plane systolic excursion; LVAW; s, left ventricular anterior wall thickness during systole; LVAW; d, left ventricular anterior wall thickness during diastole; LVPW; s, left ventricular posterior wall thickness during systole; LVPW; d, left ventricular posterior wall thickness during diastole; LVESVI, left ventricular end systolic volume index; LVEDVI, left ventricular end diastolic volume index; RVFWT, right ventricular free wall thickness. Data were analysed using two-way ANOVA with P<0.05 indicating statistical significance. Parameters were superscripted (^b^) if log-transforming the data shifted distribution closer to normal, enough to be preferable to perform statistics on, but not enough to achieve normalcy, with statistics presented as median ± IQR. Parameters were superscripted (^a^) if log-transforming the data did not help achieve normal distribution, in which case data were left untransformed and statistics were presented as median ± IQR. Normally distributed data were presented as mean ± SEM. *P<0.05 versus T1DM Mock. Bold values are statistically significant (p<0.05).

## Discussion

IAV infection has been linked with cardiac complications, however the mechanisms by which this occurs and whether this is more pronounced in patients with DM remains unclear. This is vital information for both patient management as well as future pandemic preparedness.

No significant difference in IAV severity was observed between control and T1DM mice. While this was unexpected, a prior study has reported that T1DM mice infected with either A/Phil/82(H3N2) or A/HKx31(H3N2) IAV had increased viral titers compared to non-DM mice. In contrast, no difference in A/PR/8/34 was observed, attributed to the collectin resistance and poor glycosylation of A/PR/8/34 (Reading et al., 1998).

Despite no significant difference in respiratory disease, we observed clear elevations in serum cardiac damage biomarkers in IAV-infected T1DM mice compared to the IAV-infected control group which, coupled with the observed difference in stroke volume, suggest type II ischemia. Furthermore, echocardiography revealed numerous differences in cardiac function and structure attributable to IAV or the interaction of IAV with T1DM. Preserved EF, mitral E’, LVESVI and LVEDVI indicate no obvious LV dysfunction. However, increased wall thickness and mass index suggest that hypertrophy is more severe in infected T1DM mice than in infected controls. Within the RV, IAV infection induced decreases in TAPSE, PAT and the magnitude of tricuspid E’ indicative of both systolic and diastolic dysfunction, however FAC was preserved in both control and T1DM mice. Taken together, these data indicate that IAV infection induces mild systolic and diastolic RV dysfunction in both control and T1DM mice equally, but that LV hypertrophy is more severe in T1DM mice despite preservation of function. This early development of compensative hypertrophy may be attributable for the observed increase in serum CTNI levels in IAV-infected T1DM mice and could go on to cause maladaptive remodeling (Schiattarella and Hill, 2015).

At present, the reasons for the observed increase in IAV-induced cardiac complications in T1DM are unclear. As the respiratory infection did not increase in severity with the addition of T1DM, the observed cardiac complications are unlikely to be an indirect effect of hypoxia accompanying pulmonary distress, although pulmonary embolism or micro emboli cannot be ruled out as potential contributing factors to the observed RV dysfunction upon IAV infection. Previous studies in both human patients and mice have reported a possible direct effect of IAV or viral antigens in the heart (Davoudi et al., 2012). We observed very low viral titers within the cardiac homogenate of both T1DM and control mice, suggesting this is unlikely to be the predominant driver of the observed cardiac injury in these mice, although this effect cannot be ruled out. It is important to note that the primary feature of the T1DM model is hyperglycaemia. It is therefore tempting to speculate that we are witnessing the synergistic effect of a hyperglycaemia-induced higher baseline level of inflammation within the T1DM mice even before the infection with IAV. This already-elevated inflammation may then be tipped over a ‘breaking point’ by the infection, inducing a cytokinemia that provokes microcirculation disorders and myocardial injury (Giacco and Brownlee, 2010). These cardiac complications may, alternatively, be attributable to a multitude of other effects induced by abnormal circulating substrates, and the co-existence and complex interactions with other, even subclinical, conditions in this treatment group. Importantly, in a clinical setting the contribution of underlying DM to the risk of viral cardiac complications likely depends on the duration of pre-existing disease (Giacco and Brownlee, 2010).

Together, our data aid in understanding the role of DM in virus-induced cardiac complications and may help guide clinical management of at-risk patients, while also suggesting that reducing the burden of DM in the community is not only of benefit in and of itself but it may also play an important role in reducing the extra-respiratory complications of influenza virus.

## Author’s Note

This information has previously been presented *via* a poster at the Seventh ESWI Influenza Conference and the 2020 AVS Virtual Symposium. Please direct correspondence to Dr. Kirsty Short (email: k.short@uq.edu.au; Tel.: +61 7 336 54226).

## Data Availability Statement

The original contributions presented in the study are included in the article/**Supplementary Material**. Further inquiries can be directed to the corresponding author.

## Ethics Statement

The animal study was reviewed and approved by The University of Queensland Office of Research Ethics (SBMS/071/17).

## Author Contributions

Study conception and design: KS, MAR, CB, LG, MER, and WT. Data acquisition, analysis and interpretation: JS, KS, MAR, CB, HC, KC, JR, YY, HB-O, LS, KH, NV, EN, and MW. Drafting the article: JS and KS. Article revision: JS, LG, MAR, and KS. All authors contributed to the article and approved the submitted version.

## Funding

KS was supported by the Australian Research Council [DE180100512]. The study sponsor/funder was not involved in the design of the study; the collection, analysis, and interpretation of data; writing the report; and did not impose any restrictions regarding the publication of the report.

## Conflict of Interest

The authors declare that the research was conducted in the absence of any commercial or financial relationships that could be construed as a potential conflict of interest.

## Publisher’s Note

All claims expressed in this article are solely those of the authors and do not necessarily represent those of their affiliated organizations, or those of the publisher, the editors and the reviewers. Any product that may be evaluated in this article, or claim that may be made by its manufacturer, is not guaranteed or endorsed by the publisher.
