# Supplementary material for: Type I Diabetes Mellitus Increases the Cardiovascular Complications of Influenza Virus Infection
Source: Front Cell Infect Microbiol. 2021 Sep 14;11:714440. doi: 10.3389/fcimb.2021.714440 (PMC8476859; doi:10.3389/fcimb.2021.714440)
Supplement: Supplementary file 1 [file DataSheet_1.docx]

**SUPPLEMENTARY DATA**


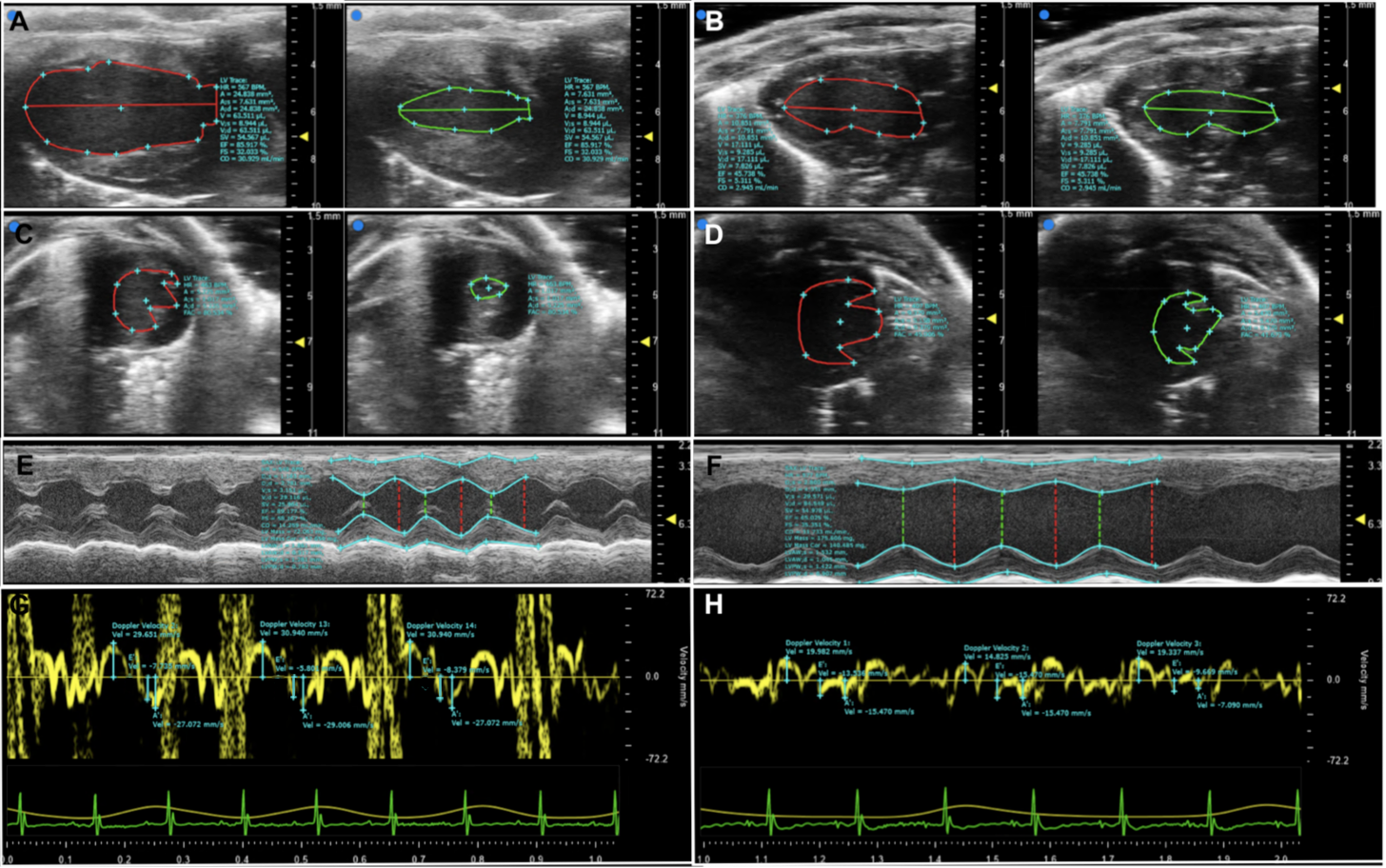


**
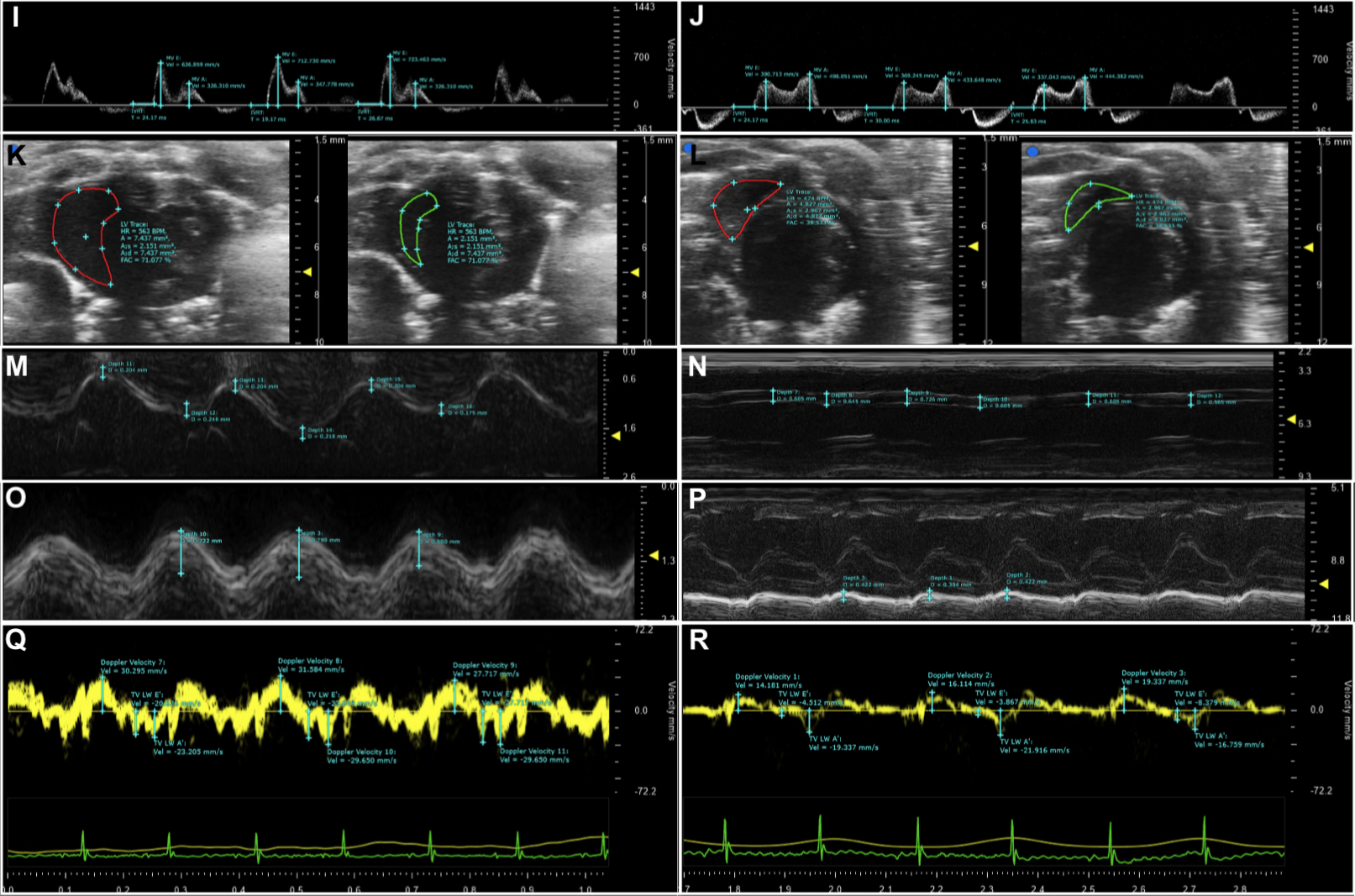
**

**
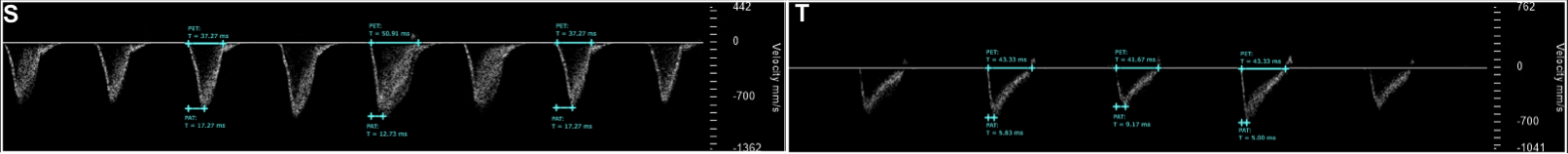
**

**Supplementary Figure 1**: Photographs exemplifying analysis methods and demonstrating the difference in echocardiogram measurements between healthy male C57BL/6J mice and IAV-infected mice with T1DM. **A** | Parasternal long-axis (PSLAX) view of the left ventricle in diastole and systole; healthy. **B** | PSLAX view of the left ventricle in diastole and systole; unhealthy. **C** | Short-axis (SAX) view of the left ventricle in diastole and systole; healthy. **D** | SAX view of the left ventricle in diastole and systole; unhealthy. **E** | SAX M-mode view of the left ventricle; healthy. **F** | SAX M-mode view of the left ventricle; unhealthy. **G** | 4-chamber view (4CV) tissue Doppler image of the mitral valve; healthy. **H** | 4CV tissue Doppler image of the mitral valve; unhealthy. **I** | 4CV pulse-wave Doppler image of blood flow through the mitral valve; healthy. **J** | 4CV pulse-wave Doppler image of blood flow through the mitral valve; unhealthy. **K** | SAX view of the right ventricle in diastole and systole; healthy. **L** | SAX view of the right ventricle in diastole and systole; unhealthy. **M** | SAX M-mode view of the right ventricle showing right ventricular free wall thickness; healthy. **N** | SAX M-mode view of the right ventricle showing right ventricular free wall thickness; unhealthy. **O** | 4CV M-mode view of depicting the tricuspid annular plane systolic excursion (TAPSE); healthy. **P** | 4CV M-mode view of depicting TAPSE; unhealthy. **Q** | 4CV tissue Doppler image of the tricuspid valve; healthy. **R** | 4CV tissue Doppler image of the tricuspid valve; unhealthy. **S** | SAX pulse-wave Doppler image of blood flow through the pulmonary artery; healthy. **T** | SAX pulse-wave Doppler image of blood flow through the pulmonary artery; unhealthy. Photographs are examples of typical ‘healthy’ and ‘unhealthy’ only and are not necessarily from the same mice; inconsistencies between photos are not reflective of actual data.

**
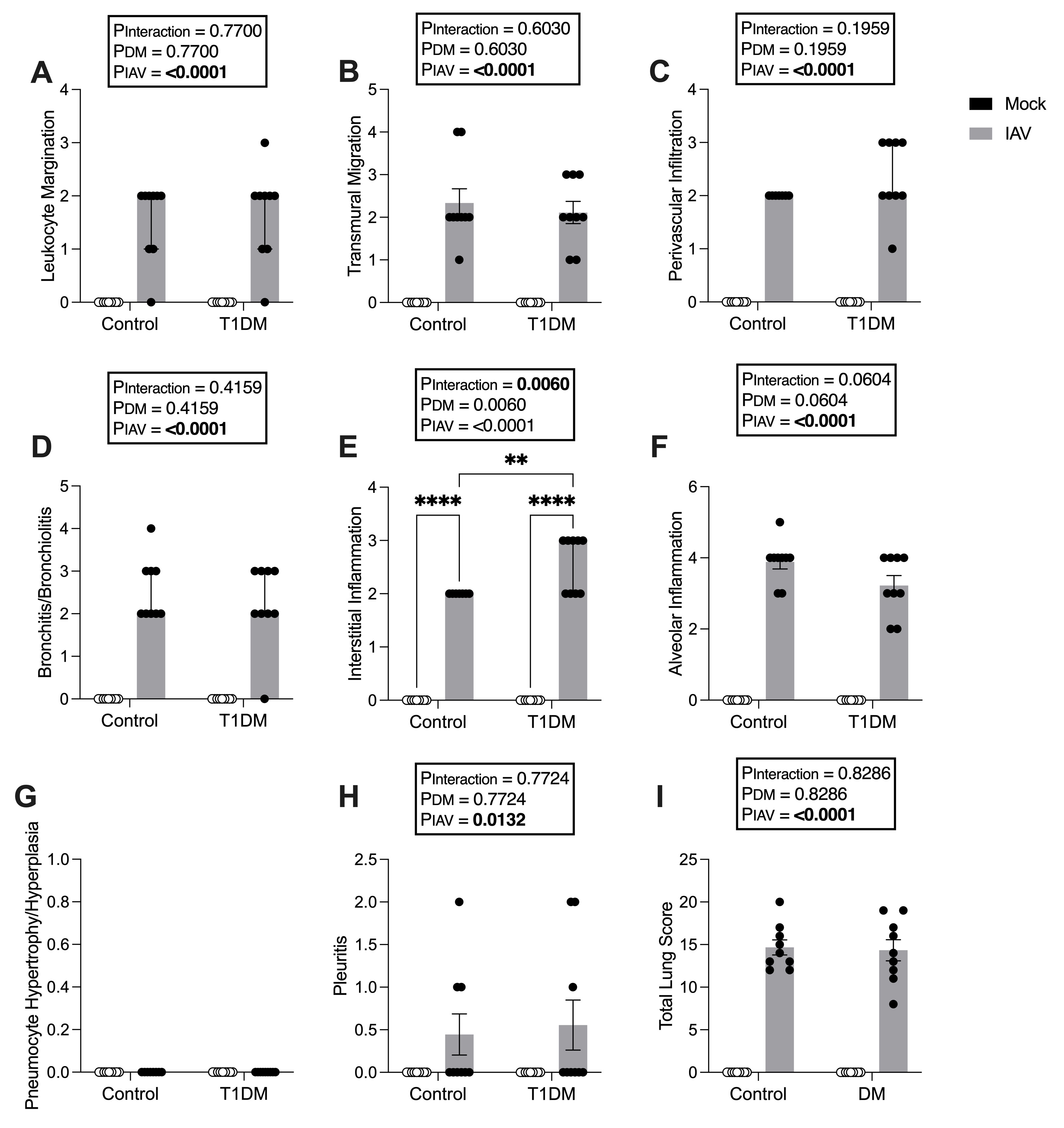
**

**Supplementary Figure 2:** Scoring of lung histology in IAV-infected male C57BL/6J mice with and without T1DM. **A** | Leukocyte margination score. **B** | Transmural migrations score. **C** | Perivascular infiltration score. **D** | Bronchitis/bronchiolitis score. **E** | Interstitial inflammation extending into alveolar septae beyond the immediate perivascular and peribronchiolar interstitial tissue score. **F** | Alveolar inflammation score. **G** | Pneumocyte hypertrophy/hyperplasia score. **H** | Pleuritis score, including mesothelial cell hypertrophy. **I** | Total lung score. Scores: 0 = no change; 1 = minimal change (or possibly non-specific background or appearance compounded by lung collapse, i.e., not inflated); 2 = mild change; 3 = moderate change; 4 = severe change in <50% of lung lobe; 5 = severe change in >50% of lung lobe. Each data point represents one mouse with at least n=9 per group and mean ± SEM for graphs B, F, G, H, and I, and median ± IQR for graphs A, C, D, and E. Statistical analysis was performed as described in “Methods” with ***P*<0.01; *****P*<0.0001.
